# Magnetic resonance imaging of post-systolic shortening in a closed-chest rat model of coronary occlusion using myocardial tagging

**DOI:** 10.1186/1532-429X-13-S1-P113

**Published:** 2011-02-02

**Authors:** Stephany Gardier, Jean-Noel Hyacinthe, Manuel Jorge-Costa, Denis R Morel, Jean-Paul Vallée

**Affiliations:** 1 Radiology Department, Faculty of Medicine, University of Geneva and Geneva University Hospita, Geneva, Switzerland; 2 Anesthesiology, Pharmacology and Intensive Care Department, Faculty of Medicine, University of Geneva and Geneva University Hospitals, Geneva, Switzerland

## 

The aim of the study was to evaluate the reliability and sensitivity of tagged cardiac magnetic resonance imaging (cMRI) in assessment of regional cardiac dysfunction induced by short ischemia.

For clinical low dose dobutamine tests, quantitative strain analyses derived from tagged cMRI have been shown to be able to detect viable myocardium. However these preliminary data were obtained in patients with high grade coronary stenosis, likely to have a large ischemic territory. So it is not known whether MRI tagging could detect coronary occlusion i) of short duration and ii) in small ischemic areas.

A model of closed-chest coronary artery occlusion was developed in Sprague-Dawley rats. 10 rats underwent a 3 minutes coronary occlusion followed by 30 minutes of reperfusion, directly in a 1.5 Tesla clinical MRI scanner. Efficiency and reversibility of the coronary occlusion was assessed by modifications of the ECG recording. Tagged images were acquired every 2.5 minutes using C-SPAMM with segmented cine fast field echo sequence. Strain analysis was performed with the Extrema Temporal Chaining algorithm in six sectors of the short axis slice, classified in 2 groups: anterolateral and inferolateral sectors were considered as ischemic whereas inferior, inferoseptal and anteroseptal sectors were remote. Contraction (maximum strain) and contractility (strain rate) derived from the myocardial strain curves were analysed using a linear model. Eight rats underwent hemodynamic measurements by intraventricular catheterism to evaluate global cardiac function along a similar protocol of ischemia-reperfusion.

During the occlusion, contraction was reduced and delayed in the ischemic sectors compared to remote group (p<0.001). The contraction remained delayed during the first minutes of reperfusion (p<0.005) in the ischemic group, demonstrating post-systolic shortening. Contractility was decreased both during the occlusion and the first minutes of reperfusion. Hemodynamic measurements dit not show any abnormality of the systolic or diastolic function after the first minutes of reperfusion.

We showed for the first time in a rat model that MRI tagging of a short coronary occlusion is feasible, on a 1.5 Tesla clinical scanner. We demonstrated in the ischemic sectors a transient contractile dysfunction which was not demonstrated by hemodynamic measurements. Besides the interest of such a method for non-invasive study of cardiac function in rodent, our data also suggest that tagged MRI could bring an important improvement in evaluation of the human cardiac function during stress imaging.

